# *Glaresishespericula* sp. n. from the Cape Verde Islands (Coleoptera, Scarabaeoidea, Glaresidae)

**DOI:** 10.3897/zookeys.792.28870

**Published:** 2018-10-23

**Authors:** David Král, Lucie Hrůzová

**Affiliations:** 1 Charles University, Faculty of Science, Department of Zoology, Viničná 7, CZ-128 43 Praha 2, Czech Republic Charles University Prague Czech Republic

**Keywords:** Afrotropical region, Coleoptera, Glaresidae, *
Glaresis
*, new species, Republic of Cabo Verde, Scarabaeoidea

## Abstract

*Glaresishespericula***sp. n.** from the Cape Verde Islands (Boa Vista Island) is described and its diagnostic characters are illustrated. The new species is compared with similar and probably closely related species *Glaresiswalzlae* Scholtz, 1983. The differential diagnosis is mainly based on the different shape of meso- and metatibiae.

## Introduction

The scarabaeoid family Glaresidae includes only the single genus *Glaresis* Erichson, 1848 widespread in all zoogeographical regions except Australia and Antarctica. The genus comprises more than eight dozen of described species of small, uniformly looking beetles that usually prefer sandy, often arid habitats. Adults are active in the evening, often attracted by light sources. The immature stages and biology of this hidden living group are not yet known (e.g., Scholtz and Grebennikov 2016). In a phylogenetical analysis based on morphology, *Glaresis* was placed as the sister taxon of the remaining Scarabaeoidea (Browne and Scholtz 1999). Fossil records of seven mesozoic glaresids are classified in three genera (*Cretoglaresis* Nikolajev, 2007; *Glaresis* and *Lithoglaresis* Nikolajev, 2007) (Bai et al. 2014). Currently 82 extant species are assigned to the genus *Glaresis* (Král and Bezděk 2016; Gordon and Hanley 2014; Král and Batelka 2017; Král et al. 2017; Paulsen 2016; Zidek 2015).

The Afrotropical fauna of the family Glaresidae is inadequately known. Only 19 species have been formally described from this region (see e.g., Scholtz 1982, 1983; Zidek 2015) and the only two comprehensive works (Petrovitz 1968; Scholtz 1983) have been published in the last century.

Recently collected *Glaresis* material from the Boa Vista Island, Cape Verde Archipelago, revealed another, undescribed species whose formal description we present below.

## Materials and methods

Specimens were examined with an Olympus SZ61 stereomicroscope, measurements were taken with an ocular grid. The habitus photographs were taken using a Canon MP-E 65mm/2.8 1–5× Macro lens attached to a Canon EOS 550D camera. Partially focused images of each specimen were combined using Zerene stacker software. Male genitalia images were taken with a Provis AX70 (Olympus) microscope with digital image processing capability using Micro Image (Olympus) software.

Specimens of the newly described species are provided with one printed red label:

“*Glaresis* | *hespericula* sp. nov. | HOLOTYPUS ♂ [or] PARATYPUS ♀ | David Král & Lucie Hrůzová 2018”. Both type specimens are deposited in the National Museum Praha, Czech Republic.

Exact label data are cited for the type material examined. Lines within each label are separated by a single vertical bar “|”. Information in quotation marks indicates the original spelling. Our remarks and additional comments are placed in brackets.

For morphological terms used in the description we largely follow Gordon and Hanley (2014) and Král et al. (2017).

## Taxonomy

### Glaresis hespericula sp. n.

Taxon classificationAnimaliaColeopteraGlaresidae

http://zoobank.org/27201324-EB3E-4B2A-902E-C26F7DBC94A9

Figures 1–10

#### Type locality.

Cape Verde, Boa Vista Island, 10 km S of Sal Rei, near Praia de Chavez 16.12°N 22.91°W, [ca. 7 m a. s. l.].

#### Type material.

Holotype (♂) and paratype (♀), “CAPE VERDE Boa Vista | Isl., 10 km S of Sal Rei, N | 16°12' W22°91'; near Praia | de Chavez, 28.-29.x.2015, | on light, V. Novák lgt.”

#### Description of male holotype.

**Figures 1–10.**
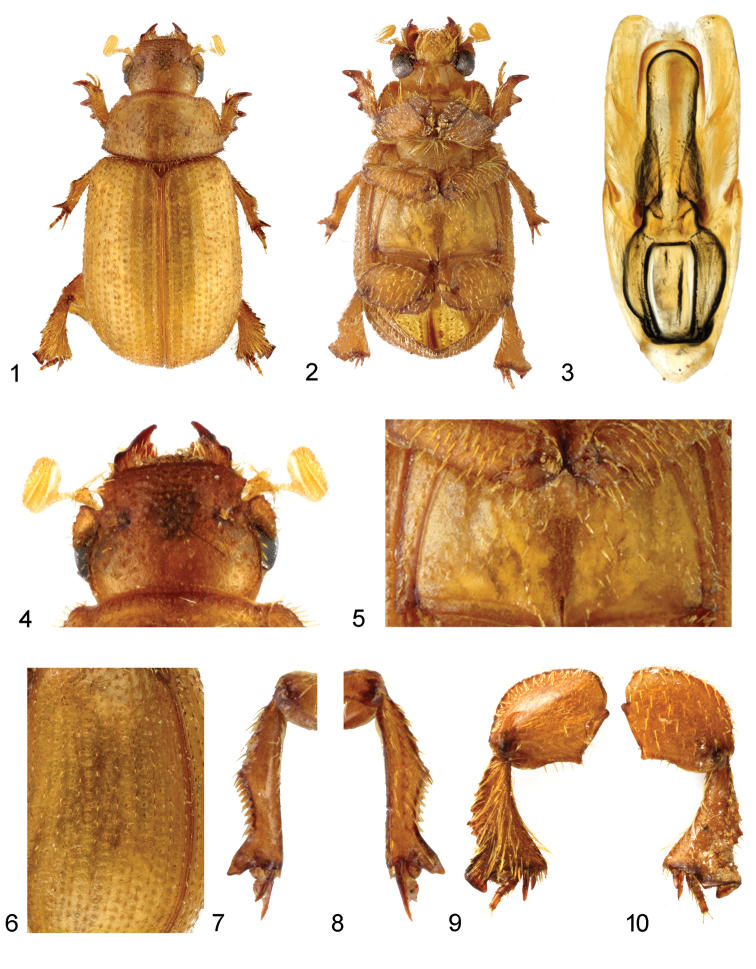
*Glaresishespericula* sp. n. **1** habitus, holotype, ♂, dorsal view **2** habitus, paratype, ♀, ventral view **3** aedeagus, dorsal view **4** head, dorsal view **5** meso-metaventral area, ventral view **6** detail of left elytron, dorsal view **7, 8** – left middle leg (**7** dorsal view, **8** ventral view) **9, 10** right hind leg (**9** dorsal view, **10** ventral view). Not to scale.

*Body* robust, strongly convex, weakly widened posteriad, brownish yellow coloured, weakly shining, macrosetation pale (Figs 1, 2).

*Head* (Figs 1, 4) surface finely rugose, semialutaceous. Mandibles robust, with strong lateral prominence, external margins sinuate. Anterior margin of clypeus shallowly sinuate, distinctly upturned, smooth, lateral angles rounded; lateral margin shallowly sinuate; posterior angles acutely angular. Surface of frons and clypeus covered with sparsely, irregularly spaced, shiny tubercles, some of them bearing stout, semi-erect macrosetae. Genae transversal, lateral margin rounded, smooth and bare. Epistomal grooves distinct. Occiput with irregularly spaced tubercles, tubercles somewhat smaller than on clypeus and frons. Each tubercle bearing very short, indistinguishable macroseta.

*Pronotum* (Figure. 1) transverse, moderately convex, pronotal grooves absent, medial longitudinal groove shallow; margins not bordered; anterolateral, lateral and basal margins serrate and with row of approximately clavate macrosetae, posterior corners rectangular; surface covered with densely almost regularly longitudinal carinae, each carina bearing thick, recumbent macroseta.

*Scutellar* plate small, almost triangular, alutaceous, smooth, and bare.

*Elytra* (Figs 1, 6) strongly convex, with ten striae and ten intervals; each stria with a row of coarse, simple punctures; intervals 1–7 and 10 remarkably costate, 8, 9 flat, all bearing a row of short, simple to weakly clavate, erect macrosetae.

Macropterous.

*Pygidium* weakly shining, scabrous.

*Ventral surface* (Figs 2, 5) alutaceous, abdominal ventrites covered with sparse fine macrosetae. Metaventral plate flat, bare and smooth, bearing row of stout macrosetae all around and with darkened translucent, longitudinal endocarina basally. Metaventral oblique grooves absent (Figure 5).

*Legs*. Posterior-superior margin of metafemora with blunt, broadly triangular teeth, anterior-superior margin of metafemora with a row of long macrosetae (Figs 9–10). Protibia distinctly tridentate (Figs 1, 2). Mesotibia (Figs 7, 8) long, nearly straight, with prominent median projection situated approximately in middle of length of mesotibia, distal part of outer edge broadly, shallowly emarginate, bearing nine short, stout spines; basal external tooth of mesotibia slightly emarginate basally. Metatibia (Figs 9, 10) broadly triangular, outer margin irregularly serrate, with faint median projection and faint median ridge, strongly macrosetaceous; row of four spine-bearing tubercles extending from base to apex medially; inner margin smooth, macrosetaceous; apex of metatibia with outer horseshoe shaped portion sub-equal than inner spur-bearing portion; inner margin of the horseshoe portion with a row of contiguous short macrosetae.

*Male external genitalia* (Figure 3). Aedeagus with parameres distinctly longer than phallobasis; parameres sclerotized in whole length, lateral margin regularly arcuate to almost regularly rounded tips; phallus sclerotised, sides straight, weakly divergent anteriad.

#### Sexual dimorphism and variability.

Female paratype differs from male by body indistinctly broader posteriad (Figure 2) and by row of ten spines on outer edge of distal part of mesotibia.

#### Measurements.

Total body length: 4.0–4.3 mm (holotype 4.2 mm; paratype 4.3 mm).

#### Differential diagnosis.

The new species is similar to *Glaresiswalzlae* Scholtz, 1983, described from Sudan, mainly in having the following characters: absence of the pronotal grooves beside the medial longitudinal groove (Figure 1), absence of the metaventral oblique grooves (Figure 5), protibia with three prominent teeth (Figs 1, 2), and smooth anteior clypeal margin (Figs 1, 4); for more details see also Petrovitz (1968) and Scholtz (1983). From this species, *Glaresishespericula* sp. n. clearly differs in the following characters:

– mesotibia with prominent median projection situated approximately in middle of length of mesotibia (Figs 1, 2, 7, 8) (mesotibia with small median projection situated before middle of length of metatibia in *G.walzlae* (Scholtz 1983: fig. 8));

– distal part of outer edge of mesotibia broadly, shallowly emarginate, with row of 9–10 spines (Figs 1, 2, 7, 8) (distal part of outer edgr of mesotibia straight, with row of 7–9 spines (Scholtz 1983: fig. 8));

– metatibia with faint median projection on outer margin, with faint median ridge (Figs 1, 2, 9, 10) (metatibia with prominent median projection on outer margin, with distinct median ridge (Scholtz 1983: fig. 19)).

#### Collecting events.

The material was collected on sand dunes using a light trap approximately between 7–9 p.m., the temperature was around 24 °C and two days before it rained very strongly.

#### Etymology.

*Hespericula* means a small, yet juvenile hesperid; noun in apposition.

#### Distribution.

So far known only from the Boa Vista Island, the Cape Verde Islands.

## Discussion

Cape Verde Archipelago is classified together with other volcanic archipelagos (Azores, Madeira, Salvagens Islands and Canary Islands) and a thin strip of the Atlantic coast in southern Portugal, Morocco and the Western Sahara as the Macaronesian biogeographic subregion (e.g., Oromí 2004). Although Cape Verde harbours a high proportion of Afrotropical species, aside from its numerous endemic taxa, its insect fauna is poor both in endemic forms and in total number of species. Their fauna is considered unique and in some aspects deserves protection (e.g., Arechavaleta et al. 2005; Batelka and Straka 2011). Fauna of Coleoptera of the individual archipelagos and islands were treated as follows: Azores (Borges et al. 2005), Madeira and related islands (Borges et al. 2008), the Salvagens Islands (Erber and Wheater 1987), the Canary Islands (Machado and Oromí 2000) and the Cape Verde Archipelago (Oromí et al. 2005). No Glaresidae have been reported from these islands and archipelagos so far. Hence, the newly described species represents the first record of the family Glaresidae from the volcanic islands west of Africa.

## Supplementary Material

XML Treatment for Glaresis hespericula
